# Supplementary material for: What Motivates the Vaccination Rift Effect? Psycho-Linguistic Features of Responses to Calls to Get Vaccinated Differ by Source and Recipient Vaccination Status
Source: Vaccines (Basel). 2023 Feb 21;11(3):503. doi: 10.3390/vaccines11030503 (PMC10057482; doi:10.3390/vaccines11030503)
Supplement: Supplementary file 1 [file vaccines-11-00503-s001.zip › vaccines-2223304-supplementary.pdf]

Supplementary 1: Complete Results

3.1. Vaccination Rift

The starting point for our linguistic analyses was the observation that vaccinated sources criticizing the unvaccinated elicited longer responses that identical comments from an unvaccinated source.[1] All linguistic measures were highly skewed, and we accordingly used non-parametric Wilcoxon tests with continuity corrections to probe the main effects of comment source on linguistic markers.

We first sought to explore the potential rift in complexity of participants' responses. Compared to those who received a comment from the unvaccinated source, participants in the vaccinated source condition communicated more elaborately, that is, used more words per sentence ($Mdn_{\text{unvaccinated source}}$ = 10.71 vs. $Mdn_{\text{vaccinated source}}$ = 11.34), *W* = 156069, *mu* = -1.00, 95%CI [-2.00; -0.17], *p* = .009, and more simply, that is used fewer words with six letters or more ($Mdn_{\text{unvaccinated source}}$ = 32.42 vs. $Mdn_{\text{vaccinated source}}$ = 31.80), *W* = 184382, *mu* = 1.62, 95%CI [0.03; 3.33], *p* = .022, and more words that were included in the LIWC2015 dictionary ($Mdn_{\text{unvaccinated source}}$ = 84.19 vs. $Mdn_{\text{vaccinated source}}$ = 85.71), *W* = 156998, *mu* = -1.25, 95%CI [-2.50; -< 0.01], *p* = .014. Apparently, participants increased their effort to make themselves understood when communicating across group lines.

The five broad summary scores provided by LIWC (Analytic, Clout, Authentic, and Tone) did not differ significantly between conditions, *W*s < 167255, *p*s > . 500. However, participants in the vaccinated source condition used more personal pronouns ($Mdn_{\text{unvaccinated source}}$ = 3.85 vs. $Mdn_{\text{vaccinated source}}$ = 4.93), *W* = 157896, *mu* < -0.01, 95%CI [-0.18; < -0.01], *p* = .019. Follow-up analyses indicated that this vaccination rift effect emerged for 3. Person pronouns addressing others ($Mdn_{\text{unvaccinated source}}$ < 0.01 vs. $Mdn_{\text{vaccinated source}}$ < 0.01), *W* = 156596, *mu* < -0.01, 95%CI [-< 0.01; -< 0.01], *p* = .002, and impersonal pronouns ($Mdn_{\text{unvaccinated source}}$ = 3.85 vs. $Mdn_{\text{vaccinated source}}$ = 5.26), *W* = 148746, *mu* = -0.14, 95%CI [-1.26; < -0.01], *p* < .001. No effect emerged for 1. Person pronouns addressing the writer themselves ("I", $Mdn_{\text{unvaccinated source}}$ < 0.01 vs. $Mdn_{\text{vaccinated source}}$ < 0.01), *W* = 168353, *mu* < -.01, 95%CI [< -.01; < .01], *p* = .593; their group ("we"; $Mdn_{\text{unvaccinated source}}$ < 0.01 vs. $Mdn_{\text{vaccinated source}}$ < 0.01, *W* = 175103, *mu* < -.01, 95%CI [< -.01; < .01], *p* = .255; or 2. Person pronouns addressing others directly ("you", $Mdn_{\text{unvaccinated source}}$ < 0.01 vs. $Mdn_{\text{vaccinated source}}$ < 0.01), *W* = 170536, *mu* < -.01, 95%CI [< -.01; < .01], *p* = .620. Apparently, the vaccination rift leads to communicating about others and things rather than with each other.

We next explored the emotional, cognitive, and motivational processes underlying the vaccination rift effect in more detail. Social conflict is commonly assumed to hinge on emotional processes with reduced cognitive processing. Contrary to this popular assumption, we did not observe a vaccination rift effect in the emotional content of the free-text responses ($Mdn_{\text{unvaccinated source}}$ = 4.74 vs. $Mdn_{\text{vaccinated source}}$ = 5.56), *W* = 160304, *mu* < -.01, 95%CI [-.54; < .01], *p* = .058. Exploratory follow-up analyses on positive emotions, negative emotions, as well as specific negative emotions (anger, anxiety, and sadness) all did not indicate a vaccination rift, *W*s < 162994, *p*s > .140. Finally, the vaccination rift also did not emerge on indicators of cognitive processes ($Mdn_{\text{unvaccinated source}}$ = 25.00 vs. $Mdn_{\text{vaccinated source}}$ = 24.90), *W* = 178236, *mu* = 0.74, 95%CI [-0.33; 2.30], *p* = .217. These findings indicate that responses to vaccination rifts may rely less on increased emotional and reduced cognitive processes that previously assumed.

Taking an alternative perspective, outgroup criticism may violate participants' basic needs. Indeed, a vaccination rift effect on motivational indicators emerged such that comments from the unvaccinated source led to using fewer drive-related words ($Mdn_{\text{unvaccinated source}}$ = 7.69) as compared to participants who received a comment from the vaccinated source ($Mdn_{\text{vaccinated source}}$ = 9.01), *W* = 159022, *mu* = -0.27, 95%CI [-1.41; < -0.01], *p* = .035. Follow-up analyses indicated that this effect especially emerged for achievement-related words ($Mdn_{\text{unvaccinated source}}$ = < 0.01, $Mdn_{\text{vaccinated source}}$ = 1.73), *W* = 156967, *mu* < -0.01, 95%CI [< -0.01; < -0.01], *p* = .009. No effects emerged on words related to affiliation, power, risk, or reward, all *W*s < 163328, all *p*s > .150. Apparently, the vaccination rift effect hinges on

perceptions of one's threatened competence and achievement more than affiliation or shifting risk-perceptions.

Finally, exploratory analyses on perception processes, biological processes, time focus, relativity and informal language did not show any effects, all Ws < 160679, all ps > .059. Responses to the vaccinated source referred more to social processes ($Mdn_{\text{unvaccinated source}}$ = 9.09, $Mdn_{\text{vaccinated source}}$ = 10.09), W = 153742, mu = -0.74, 95%CI [-2.11; < -0.01], p = .002, but no effects on the subcategories family, friends, female, and male emerged in follow-up analyses, all Ws < 166474, all ps > .110.

3.2. Participant Vaccination Status

Our next goal was to test whether responses varied based on participants' own vaccination status, collapsed across message source conditions. To this end, we clustered participants into two vaccination status groups: Vaccinated (1, 2, or 3 doses, n = 634) and unvaccinated (recovered or unvaccinated, n = 536). For exploratory purposes, we also compared the key subgroups of fully vaccinated (3 doses; n = 466) and unvaccinated participants (n = 370). Results between the full sample and this subsample did not differ, unless noted otherwise.

We first sought to explore the length and complexity of participants' responses. Compared to those who indicated that they were vaccinated, unvaccinated participants wrote more ($Mdn_{\text{unvaccinated participant}}$ = 31.50 vs. $Mdn_{\text{vaccinated participant}}$ = 15.00), W = 226893, mu = 14.00, 95%CI [11.00; 17.00], p < .001, and used more words per sentence ($Mdn_{\text{unvaccinated participant}}$ = 12.43 vs. $Mdn_{\text{vaccinated participant}}$ = 10.00), W = 205690, mu = 2.50, 95%CI [1.71; 3.17], p < .001. Unvaccinated participants used fewer words with six letters or more ($Mdn_{\text{unvaccinated participant}}$ = 30.93 vs. $Mdn_{\text{vaccinated participant}}$ = 33.33), W = 153199, mu = -2.20, 95%CI [-3.70; -0.59], p < .001. No effect emerged on the use of words included in the dictionary ($Mdn_{\text{unvaccinated participant}}$ = 84.48 vs. $Mdn_{\text{vaccinated participant}}$ = 85.71), W = 160938, mu = -0.69, 95%CI [-1.93; < 0.01], p = .119. However, when excluding partly vaccinate and recovered participants, a small effect emerged. Those unvaccinated participants used somewhat fewer dictionary words than those fully vaccinated ($Mdn_{\text{unvaccinated participants only}}$ = 83.99 vs. $Mdn_{\text{fully vaccinated participants}}$ = 85.71), W = 78999, mu = -1.36, 95%CI [-2.93; < -0.01], p = .037. Apparently, in comparison to vaccinated participants, those unvaccinated responded more to the call to get vaccinated and used somewhat simpler words.

We next analyzed the five broad summary scores provided by LIWC. Unvaccinated participants used language that was less analytic ($Mdn_{\text{unvaccinated participant}}$ = 37.27 vs. $Mdn_{\text{vaccinated participant}}$ = 64.06), W = 146281, mu = -2.41, 95%CI [-7.24; < -0.01], p < .001, less confident ($Mdn_{\text{unvaccinated participant}}$ = 45.33 vs. $Mdn_{\text{vaccinated participant}}$ = 61.77), W = 144220, mu = -6.94, 95%CI [-10.57; -3.37], p < .001, and less positive in tone ($Mdn_{\text{unvaccinated participant}}$ = 17.39 vs. $Mdn_{\text{vaccinated participant}}$ = 17.39), W = 140151, mu = < -0.01, 95%CI [-0.61; < -0.01], p < .001. However, messages by unvaccinated participants used more authentic language ($Mdn_{\text{unvaccinated participant}}$ = 41.77 vs. $Mdn_{\text{vaccinated participant}}$ = 16.53), W = 186943, mu = < 0.01, 95%CI [< 0.01; 3.29], p = .003. These observations comport well with the assumption that calls to get vaccinated represent a personal threat to those unvaccinated, independent of the source these calls come from.

Unvaccinated participants used more personal pronouns ($Mdn_{\text{unvaccinated participant}}$ = 4.79 vs. $Mdn_{\text{vaccinated participant}}$ = 3.96), W = 185189, mu < 0.01, 95%CI [< 0.01; 0.65], p = .006. This effect emerged for 3. Person pronouns addressing others ($Mdn_{\text{unvaccinated participant}}$ = 0 vs. $Mdn_{\text{vaccinated participant}}$ = 0), W = 201344, mu < 0.01, 95%CI [< 0.01; < 0.01], p < .001, and impersonal pronouns ($Mdn_{\text{unvaccinated participant}}$ = 6.25 vs. $Mdn_{\text{vaccinated participant}}$ = 1.98), W = 223006, mu = 2.94, 95%CI [2.22; 3.57], p < .001. No effect emerged for 1. Person pronouns addressing the writer themselves ("I", $Mdn_{\text{unvaccinated participant}}$ = 0 vs. $Mdn_{\text{vaccinated participant}}$ = 0), W = 178054, mu < .01, 95%CI [< -.01; < .01], p = .113; their group ("we"; $Mdn_{\text{unvaccinated participant}}$ = 0 vs. $Mdn_{\text{vaccinated participant}}$ = 0, W = 171447, mu < -.01, 95%CI [< -.01; < .01], p = .255; or 2. Person pronouns addressing others directly ("you", $Mdn_{\text{unvaccinated participant}}$ = 0 vs. $Mdn_{\text{vaccinated participant}}$ = 0, W = 169724, mu < -.01, 95%CI [< -.01; < .01], p = .871. In sum, unvaccinated participants wrote more about others and things but did not address others more.

We next explored the emotional, cognitive, and motivational expressions in more detail. In line with the observations regarding emotional tone, the free-text responses by unvaccinated participants contained more negative emotion words ($Mdn_{\text{unvaccinated participant}}$ = 1.68 vs. $Mdn_{\text{vaccinated participant}}$ = 0), *W* = 205981, *mu* < .01, 95%CI [< .01; < .01], *p* < .001, and fewer positive emotion words ($Mdn_{\text{unvaccinated participant}}$ = 1.94 vs. $Mdn_{\text{vaccinated participant}}$ = 2.27), *W* = 156518, *mu* < -.01, 95%CI [< -.01; < -.01], *p* < .001. Follow-up analyses observed these differences on all three specific negative emotions anger ($Mdn_{\text{unvaccinated participant}}$ = 0 vs. $Mdn_{\text{vaccinated participant}}$ = 0), *W* = 185940, *mu* < .01, 95%CI [< .01; < .01], *p* < .001, anxiety ($Mdn_{\text{unvaccinated participant}}$ = 0 vs. $Mdn_{\text{vaccinated participant}}$ = 0), *W* = 196684, *mu* < .01, 95%CI [< .01; < .01], *p* < .001, and sadness ($Mdn_{\text{unvaccinated participant}}$ = 0 vs. $Mdn_{\text{vaccinated participant}}$ = 0), *W* = 190212, *mu* < .01, 95%CI [< .01; < .01], *p* < .001. Those unvaccinated thus feel strongly about the topic of vaccinations.

Finally, no effects of participant vaccination status emerged on indicators of cognitive processes ($Mdn_{\text{unvaccinated participant}}$ = 24.00 vs. $Mdn_{\text{vaccinated participant}}$ = 24.20), *W* = 167697, *mu* < 0.01, 95%CI [-1.67; 1.01], *p* = .701, or drive-related words ($Mdn_{\text{unvaccinated participant}}$ = 8.27, $Mdn_{\text{vaccinated participant}}$ = 8.33), *W* = 165201, *mu* < -0.01, 95%CI [-0.73; < 0.01], *p* = .410. However, exploratory follow-up analyses indicated small effects on specific motive dimensions such that unvaccinated participants used words that related more to achievement ($Mdn_{\text{unvaccinated participant}}$ = 1.91, $Mdn_{\text{vaccinated participant}}$ = 0), *W* = 186730, *mu* < -0.01, 95%CI [< -0.01; < -0.01], *p* = .002, power ($Mdn_{\text{unvaccinated participant}}$ = 1.85, $Mdn_{\text{vaccinated participant}}$ = 0), *W* = 186537, *mu* < 0.01, 95%CI [< 0.01; < 0.01], *p* = .002, and risk ($Mdn_{\text{unvaccinated participant}}$ = 0, $Mdn_{\text{vaccinated participant}}$ = 0), *W* = 191210, *mu* < 0.01, 95%CI [< 0.01; < 0.01], *p* = .002. No effects emerged on words related to affiliation or reward, all *W*s < 176606, all *p*s > .080. Overall, threatened needs seem less important for effects of vaccination status than for the vaccination rift effect, although those unvaccinated did refer somewhat more frequently to the topics of risk, power, and achievement. Interestingly, no such effect was observed for affiliation.

Exploratory analyses indicated that unvaccinated participants referred more to biological processes ($Mdn_{\text{unvaccinated participant}}$ = 3.26, $Mdn_{\text{vaccinated participant}}$ = 0), *W* = 190415, *mu* < 0.01, 95%CI [< 0.01; < 0.01], *p* < .001. Follow-up analyses showed that this was particularly true for words related to body, ($Mdn_{\text{unvaccinated participant}}$ = 0, $Mdn_{\text{vaccinated participant}}$ = 0), *W* = 197774, *mu* < 0.01, 95%CI [< 0.01; < 0.01], *p* < .001, and health, ($Mdn_{\text{unvaccinated participant}}$ = 0, $Mdn_{\text{vaccinated participant}}$ = 0), *W* = 204688, *mu* < 0.01, 95%CI [< 0.01; < 0.01], *p* < .001. No effects on words regarding ingestion or sexuality emerged after adjusting the alpha-level (.05/4 = .0125), all *W*s < 174767, all *p*s > .044. Unvaccinated participants also referred more to perception ($Mdn_{\text{unvaccinated participant}}$ = 0, $Mdn_{\text{vaccinated participant}}$ = 0), *W* = 183896, *mu* < 0.01, 95%CI [< 0.01; < 0.01], *p* = .002, especially seeing ($Mdn_{\text{unvaccinated participant}}$ = 0, $Mdn_{\text{vaccinated participant}}$ = 0), *W* = 182196, *mu* < 0.01, 95%CI [< 0.01; < 0.01], *p* < .001. Effects for hearing, *W* = 176844, *p* = .009, were non-significant when only considering those fully vaccinated or unvaccinated, *W* = 88900, *p* = .076, and effects for feeling were non-significant when correcting the alpha-level (.05/4 = .0167), *W* = 176300, *p* = .030.

Unvaccinated participants used more informal language ($Mdn_{\text{unvaccinated participant}}$ = 0, $Mdn_{\text{vaccinated participant}}$ = 0), *W* = 185436, *mu* < 0.01, 95%CI [< 0.01; < 0.01], *p* < .001. Follow-up tests did not identify reliable effects in the subcategories assent, swearwords, netspeak, nonfluencies, or fillers (an effect on assent in the full sample *W* = 176277, *p* = .006, was not significant in the subset after correcting the alpha-level (.05/5 = .01), *W* = 88953, *p* = .035). Unvaccinated participants used more relativity words ($Mdn_{\text{unvaccinated participant}}$ = 11.97, $Mdn_{\text{vaccinated participant}}$ = 10.27), *W* = 184706, *mu* = 0.42, 95%CI [< 0.01; 1.83], *p* = .009, including those related to motion ($Mdn_{\text{unvaccinated participant}}$ = 0, $Mdn_{\text{vaccinated participant}}$ = 0), *W* = 192272, *mu* < 0.01, 95%CI [< 0.01; < 0.01], *p* < .001, and space ($Mdn_{\text{unvaccinated participant}}$ = 6.89, $Mdn_{\text{vaccinated participant}}$ = 4.76), *W* = 193104, *mu* = 0.50, 95%CI [< 0.01; 1.67], *p* < .001. This difference emerged in relation to time ($Mdn_{\text{unvaccinated participant}}$ = 3.88, $Mdn_{\text{vaccinated participant}}$ = 2.07), *W* = 181884, *mu* < 0.01, 95%CI [< 0.01; < 0.01], *p* = .030, but was not significant in the subsample of only full or unvaccinated participants, *W* = 92426, *p* = .060. Unvaccinated participants used more words related to social settings ($Mdn_{\text{unvaccinated participant}}$ = 10.64, $Mdn_{\text{vaccinated participant}}$ = 8.84), *W* = 193390, *mu* = 1.68, 95%CI [0.30; 3.02], *p* < .001, especially male words ($Mdn_{\text{unvaccinated participant}}$

= 0, $Mdn_{\text{vaccinated participant}}$ = 0), W = 187960, *mu* < 0.01, 95%CI [< 0.01; < 0.01], p < .001. No effects emerged for female, family, and friends words, all *W*s < 173528, all *p*s > .180.

3.3. Matching of Participant and Source Vaccination Status

A final possibility is that the congruence between participant and source vaccination status influences the psycho-linguistic processes in responses to calls to get vaccinated. We explored this possibility by coding whether participant and source status matched (unvaccinated source & unvaccinated participant, vaccinated source & vaccinated participant, n = 564) or not (unvaccinated source & vaccinated participant, vaccinated source & unvaccinated participant, n = 606). Note that this comparison is akin to an omnibus interaction test in parametric analyses (e.g., ANOVA).

We observed no effects of this matching variables on any of the dependent measures, *W*s < 181032, *p*s > .075, with the following two exceptions: Participants whose vaccination, status matched the source used more words that were in the LIWC dictionary (Mdn_{match} = 85.50, Mdn_{mismatch}= 84.04), W = 183475, *mu* = 1.07, 95%CI [< 0.01; 2.34], p = .029, and more words related to anger (Mdn_{match} = 0, Mdn_{mismatch} = 0), W = 178651, *mu* < 0.01, 95%CI [< 0.01; < 0.01], p = .018. When only including fully vaccinated and unvaccinated participants, small effects on the summary measure of analytic language (Mdn_{match} = 52.10, Mdn_{mismatch} = 55.60), W = 94556, *mu* < 0.01, 95%CI [< -0.01; < 0.01], p = .028, and on "we" personal pronouns emerged (Mdn_{match} = 0, Mdn_{mismatch} = 0), W = 81712, *mu* < -0.01, 95%CI [< -0.01; < 0.01], p = .028, and the observed differences on dictionary words and anger were no longer significant, *W*s < 90787, *p*s > .050.

References

1. Thürmer JL, McCrea SM. The vaccination rift effect provides evidence that source vaccination status determines the rejection of calls to get vaccinated. *Scientific Reports* **12**, 18947 (2022).
